# Wondering Awe as a Perceptive Aspect of Spirituality and Its Relation to Indicators of Wellbeing: Frequency of Perception and Underlying Triggers

**DOI:** 10.3389/fpsyg.2021.738770

**Published:** 2021-09-30

**Authors:** Arndt Büssing

**Affiliations:** ^1^Professorship Quality of Life, Spirituality and Coping, Faculty of Health, Witten/Herdecke University, Herdecke, Germany; ^2^IUNCTUS – Competence Center for Christian Spirituality, Philosophical-Theological Academy, Münster, Germany

**Keywords:** awe perceptions, spirituality, mindfulness, wellbeing, secular concepts, qualitative analyses

## Abstract

**Background:** Spirituality is a multidimensional construct which includes religious, existentialistic, and relational issues and has different layers such as faith as the core, related attitudes and conviction, and subsequent behaviors and practices. The perceptive aspects of spirituality such as wondering awe are of relevance for both, religious and non-religious persons. These perceptions were related to perceiving the Sacred in life, mindful awareness of nature, others and self, to compassion, meaning in life, and emotional wellbeing. As awe perceptions are foremost a matter of state, it was the aim (1) to empirically analyze the frequency of wondering awe perceptions (i.e., with respect to gender, age cohorts, religious or non-religious persons) and (2) to qualitatively analyze a range of triggers of awe perceptions.

**Methods:** Data from 7,928 participants were analyzed with respect to the frequency of Awe/Gratitude perceptions (GrAw-7 scale), while for the second part of the study responses of a heterogeneous group of 82 persons what caused them to perceive moments of wondering awe were analyzed with qualitative content analysis techniques.

**Results:** Persons who experience Awe/Gratitude to a low extend were the youngest and had lowest wellbeing and lowest meditation/praying engagement, while those with high GrAw-7 scores were the oldest, had the highest wellbeing, and were more often meditating or praying (*p*<0.001). Gender had a significant effect on these perceptions, too (Cohen’s *d*=0.32). In the qualitative part, the triggers can be attributed to four main categories, *Nature*, *Persons*, *Unique Moments*, and *Aesthetics*, *Beauty*, and *Devotion*. Some of these triggers and related perceptions might be more a matter of admiration than wondering awe, while other perceptions could have more profound effects and may thus result in changes of a person’s attitudes and behaviors.

**Conclusion:** Emotionally touching experiences of wondering awe may result in feelings of interconnectedness, prosocial behavior, mindful awareness, and contribute to a person’s meaning in life and wellbeing and can also be a health-relevant resource. These perceptions can be seen as a perceptive aspect of spirituality, which is not exclusively experienced by religious people but also by non-religious persons.

## Introduction

Spirituality is a complex and multidimensional construct which includes religious, existentialistic, prosocial-humanistic, and relational issues (Zwingmann et al., 2011) and, from a conceptual point of view, has different layers such as some kind of “faith” as the core, related attitudes and convictions, and subsequent behaviors and practices (Büssing, 2019). Depending on the religious and cultural background, there are several definitions what spirituality is or might be (Emmons, 2000; Tanyi, 2002; Hill and Pargament, 2003; Engebretson, 2004; Büssing, 2019). Some refer to specific and exclusive religious faith traditions and only rarely cover also non-religious approaches. Other refer mainly on cognitive approaches (attitudes and convictions) and spiritual practices (praying, meditation) and less on perceptive/experiential aspects (Zwingmann et al., 2011; Büssing, 2019). However, also persons who would not regard themselves as religious may have interest in (non-religious) spiritual issues, that is, they are in search of meaning in life, something that gives their live value and orientation, they try to mindfully encounter the world around, to compassionately care for others, and they have feelings of wondering awe in specific situations (Büssing et al., 2009, 2013, 2014; Büssing, 2020). Also for children’s spirituality, it was stated that “Spirituality is the experience of the sacred other, which is accompanied by feelings of wonder, joy, love, trust, and hope (…)” (Engebretson, 2004). Moments of wonder or wondering awe are a perceptional aspect of spirituality which is experienced by various groups of persons – also by persons with special needs and cognitive impairment (Büssing et al., 2017). Although it is not an identical construct but may be related, training of mindful awareness in palliatively treated cancer patients enhanced their wellbeing and helped them to reconnect “with their values and spiritual beliefs” (Poletti et al., 2019). In patients with epilepsy, training of mindful awareness raised acceptance of their health condition and “re-integrating their condition” (Bauer et al., 2019). In line with this, both mindfulness training and gratitude interventions can improve wellbeing and mental health (O’Leary and Dockray, 2015). This finding is of relevance as gratitude, which was found to be associated with awe perceptions (Büssing et al., 2014, 2018a), mediates the link between mindfulness and positive mood states (Mirnics et al., 2020).

Feelings of wondering awe are foremost an emotional reaction toward touching experiences (Keltner and Haidt, 2003; Pearsall, 2007; Silvia et al., 2015) that do not necessarily require a religious background, although awe can be a religious experience, too (James, 1997). Gallagher et al. (2015) differentiated *awe* as a first-order level of experience from *wondering* which is described as a reflective second-order experience; they considered awe as an immediate experience that may motivate more reflective experiences. Empirical studies have shown that pausing in specific situations with feelings of wondering awe (and subsequent feelings of gratitude) is of relevance for both, religious and non-religious persons; all may perceive moments of wondering awe or admiration, yet to differentiated degrees (Büssing et al., 2013, 2014, 2020a; Büssing, 2020). Persons with depressive diseases experience awe and related feelings of gratitude similarly like other persons with psychiatric or neurological diseases, too, but they perceive the beauty of life and nature less often, which can be a trigger of feelings of awe (Büssing et al., 2014).

Yaden et al. (2019) operationalized six main dimensions of awe as a state: altered time perception, self-diminishment, connectedness, perceived vastness, physical sensations, and need for accommodation. In fact, there might be small moments of wonder or admiration, but also the more rare vast experiences that change a person’s life with a need for accommodation as suggested by Keltner and Haidt (2003). Our group’s operationalization refers more to the (phenomenologically inspired) experiential aspects (in terms of a state) that do not require altered time perceptions or feelings of vastness or explicit physical sensations. Instead, the Awe/Gratitude scale (GrAw-7; Büssing et al., 2018a) refers to the general ability to experience nature’s beauty, the perception of being captivated by the beauty of nature, to pause “spellbound at the moment” (which implies that time “stands still” for a moment), becoming “quite and devout” in specific situations and locations, and thus feelings of “wondering awe,” and subsequently feelings of gratitude because in these moments of pausing one may consider so many things one may feel grateful for. Although, the experience of vastness was seen as central by Keltner and Haidt (2003), it is a very exceptional experience which is not shared by too many persons. In most situations, awe is further not a matter of self-diminishing or even fear anymore, but a positive experience of being “touched” (Shiota et al., 2007).

In terms of positive psychology, awe perceptions are related rather to emotional well-being (Krause and Hayward, 2015; Büssing et al., 2021a), prosocial behaviors (Piff et al., 2015) and commitment to the creation and disadvantaged persons (Büssing et al., 2018b) and to mindful awareness of nature, others, and self (Büssing et al., 2020a, 2021a). Experimental studies by Rudd et al. (2012) approved that awe experiences may facilitate intentions to invest time helping others and that awe sensitizes for the “present moment,” resulting in higher life satisfaction. The underlying personality traits seem to be relevant too, as awe is related to openness to new experiences (Silvia et al., 2015; Yaden et al., 2019) and much weaker also to Neuroticism and Agreeableness (Yaden et al., 2019); it is also related to the spiritual background of a person, as awe was found to be strongly related to the intention to live from the faith and to experience the Sacred in life (Büssing et al., 2018b) and to their frequency of meditation or praying (Büssing et al., 2021a).

During the COVID-19 pandemic, Awe/Gratitude was the best predictor of perceived positive changes in terms of post-traumatic growth and a resource to recognize the still positive aspects in life (Büssing et al., 2021a,b). Using the three-item precursor version of the GrAw-7 scale, Gratitude/Awe was moderately inversely related to depressive symptoms in patients with fibromyalgia, but not with anxiety or general physical health (data source: Offenbaecher et al., 2013). Other studies found that awe may buffer negative feelings (Koh et al., 2017; Atamba, 2019) and is related to positive emotions and less anxiety (Rankin et al., 2019).

Thus, awe perceptions may sensitize people to be more aware of the world around in terms of mindfulness, to actively help others, and with emotional wellbeing and mental stability. In fact, dispositional awe was found to be related to meaning in life and subjective well-being of young adults from China, where meaning in life seems to mediate the interaction between awe and wellbeing (Zhao et al., 2019). In a study by Aschoff (2021) among medical doctors working voluntarily for people in need, Awe/Gratitude was moderately related to the presence component of meaning in life, but not with its search component. This would further indicate that meaning in life (which is one of the many aspects of spirituality) is a stabilizing factor for a person’s wellbeing that would either facilitate awe perceptions or is its outcome.

However, findings also indicate that awe perceptions may be either mild and brief in terms of an aesthetic fascination, or profound, intense, memorable, and “transformative” (Cohen et al., 2010; Silvia et al., 2015). This transformative aspect of awe, which may start with a change of views, attitudes, and eventually also behaviors and may imply responsibility taking for the world around, may link it again to the topic of spirituality (Büssing et al., 2018b). Cohen et al. (2010) described that these “highly emotional experiences” may result in “long-lasting and meaningful changes in personality” and referred these changes to the topic of “spiritual transformation,” as described by others, too (Keltner and Haidt, 2003; Ironson and Kremer, 2009; Kremer and Ironson, 2009; Büssing et al., 2018b; Penman, 2021). However, one may assume that only the profound and intense perceptions may change a person’s views, attitudes, and behaviors in terms of a “transformation,” but not necessarily the aesthetic fascination.

As perceptions of awe are foremost a matter of state (Büssing et al., 2018a; Yaden et al., 2019), it is important to analyze who is experiencing it and what the triggers are. Aim was therefore (1) to empirically analyze the frequency of wondering awe perceptions, particularly with respect who is experiencing it stronger than others (i.e., women and men, age cohorts, religious or non-religious persons, and persons with specific lifestyles) and (2) to qualitatively analyze a range of triggers of awe perceptions reported by a heterogeneous sample of participants.

## Materials and Methods

### Empirical Approach

#### Participants

For the empirical part, data sets of previous and current anonymous surveys were combined (i.e., Büssing et al., 2018a,b, 2020a, 2021a,b,c,d). Participants were informed about the study purposes, guaranteed confidentially, and they consented to participate by filling the anonym questionnaires, which were applied in most cases online. Neither identifying personal details nor IP addresses were recorded to guarantee anonymity.

#### Measures

##### Awe and Gratitude

Wondering awe is a perceptive aspect of spirituality that is experienced also by non-religious persons (Büssing et al., 2018a). To address times of pausing for astonishment or “wonder” in specific situations (mainly in the nature), perceived awe and subsequent feelings of gratitude were measured with the seven-item Awe/Gratitude scale (GrAw-7; Büssing et al., 2018a). This scale has good psychometric properties (Cronbach’s alpha=0.82) and is not contaminated with specific religious or spiritual terminology. It uses items such as “I stop and then think of so many things for which I’m really grateful,” “I stop and am captivated by the beauty of nature,” “I pause and stay spellbound at the moment,” and “In certain places, I become very quiet and devout.” Thus, Awe/Gratitude operationalized in this way is a matter of an emotional reaction toward an immediate and “captive” experience. The frequency of these perceptions was scored on a four-point scale (0 – never; 1 – seldom; 2 – often; and 3 – regularly) and finally transferred to a 100-point scale.

##### Perception of the Sacred

The Daily Spiritual Experience Scale (DSES) was developed as a measure of a person’s perception of the Sacred in daily life, and thus, the items measure experience rather than particular beliefs or behaviors (Underwood and Teresi, 2002; Underwood, 2011). In contrast to the GrAw-7 questionnaire, responding positively to the DSES-6 requires belief in God. Here, the six-item version (DSES-6; Cronbach’s alpha=0.91) was used which uses specific items such as feeling God’s presence, God’s love, desire to be closer to God (union), finding strength/comfort in God, and being touched by beauty of creation (Underwood and Teresi, 2002). The response categories from 1 to 6 are “many times a day,” “every day,” “most days,” “some days,” “once in a while,” and “never/almost never.” Item scores were finally summed up, and thus, the scores range from 6 to 36.

##### Wellbeing

To assess participants’ well-being, the WHO-Five Well-being Index (WHO-5) was used (Bech et al., 2013). Representative items are “I have felt cheerful and in good spirits” or “My daily life has been filled with things that interest me.” Respondents assess how often they had the respective feelings within the last 2weeks, ranging from “at no time” (0) to “all of the times” (5). Here, the sum scores ranging from 0 to 25 were reported.

##### Frequency of Meditation and Praying

The frequency of participants’ spiritual/religious practices such as meditation or praying was assessed with a 4-grade scale ranging from “never” (0) to “at least once per month” (1), “at least once per week” (2), and “at least once per day” (3) as described (Büssing et al., 2020b).

#### Statistical Analyses

Data of 7,928 participants were used to assess their Awe/Gratitude perceptions. Within this larger sample, different subsamples recruited as different cohorts and at different time points were analyzed with respect to participants’ wellbeing (WHO-5; *n*=5,395), frequency of spiritual practices (*n*=3,385), and perception of the Sacred (DSES-6; *n*=2,620).

Descriptive statistics, ANOVA, and first-order correlations (Spearman rho) as well as internal consistency (Cronbach’s coefficient α) were computed with SPSS 23.0. The significance level of ANOVA and correlation analyses was set at *p*<0.01. With respect to classifying the strength of the observed correlations, *r*>0.5 is regarded as a strong correlation, r between 0.3 and 0.5 as a moderate correlation, r between 0.2 and 0.3 as a weak correlation, and *r*<0.2 as negligible or no correlation.

### Qualitative Approach

#### Data Analysis

For this study, free-text responses were analyzed using qualitative content analysis techniques (Krippendorff, 2004) referring to a phenomenologically inspired approach (Neubauer et al., 2019; Zahavi, 2019). Aim was to describe what was perceived by the participants and how (Teherani et al., 2015). To get open, experience-oriented narratives, participants were invited to state situations where they experienced moments of wondering awe (“Can you please describe when or where you had such feelings of wondering awe – what were the triggers/reasons?”) and what they subsequently perceived and noticed [“What did you feel about it? (Please try to describe these feelings/sensations/reactions)”]. These free-text answers were then coded according to the mentioned topics (meaningful text segments), and a set of codes was developed. These codes were combined in a code list and grouped according to their motifs into main codes and subcodes. Representative text passages served as anchor quotations for the respective codes (categories). For this analysis, the focus is the triggers of awe which are described in detail, while the subsequent perceptions will be reported in detail elsewhere.

#### Participants

To get some degree of heterogeneity, healthy participants, both female and male, from different age cohorts and with different religious/spiritual backgrounds (incl. religious orders and yoga practitioners, but also persons without explicit spiritual practices and further non-religious persons) were invited by email in a larger European research network (i.e., university researchers, students, members of religious orders, and yoga practitioners) and subsequently informed about the purpose of the project, voluntary participation, and usage of short anonymous quotes for a publication. Eighty-two persons send back their written statements. Identifying information and respective mails were immediately deleted to assure anonymity of responses. Nevertheless, their gender, age, and religious background (which was not part of the analysis) were recorded to describe the sample. Among them, 56 were female and 26 were male, with a mean age of 52.9±13.3years (range 29–82). Thirty-three were Catholics and 21 Protestants, and 28 had other spiritual/religious backgrounds (incl. Buddhists and Yoga practitioners).

## Results

### Empirical Approach

#### Description of Participants

For this study, data from 7,928 participants were used (Table 1). Among them, 64% were women and 36% men; <1% did not state their gender. Their mean age was 46±16years. Most were nominally Christians (81%), 3% had other religious affiliations, and 16% had none. There were further two specific subsamples, one with yoga practitioners (11%) and one with religious brothers and sisters (5%). Within the whole sample, 52% were never meditating, 14% at least once per month, 17% once per week, and 17% once per day, while 45% were never praying, 12% at least once per month, 13% once per week, and 31% once per day (Table 1). Participants’ perception of the Sacred in daily life and also their wellbeing scored in the respective mid-ranges (Table 1).

**Table 1:** Sociodemographic data of participants (*N*=7,928).

	*n*	% of responders	mean±SD
Gender			
Women	5,061	64.4	
Men	2,794	35.6	
No information	73		
Age (years)	1,261		46.0±16.4
Age cohorts			
<21years	640	8.2	
21–30years	1,200	15.3	
31–40years	952	12.1	
41–50years	1,403	17.9	
51–60years	2,180	27.8	
61–70years	1,054	13.4	
>70years	412	5.2	
No information	87		
Religious affiliation			
Christians	5,789	81.1	
Others	201	2.8	
None	1,150	16.1	
No information	788		
Frequency of spiritual practices			
Meditation	3,385		0.99±1.17
Praying	3,379		1.30±1.31
Perception of the sacred (DSES-6)	2,620		22.3±7.4
Wellbeing (WHO-5)	5,395		46.0±16.4

#### Perceptions of Wondering Awe in the Sample

Participants experienced the beauty in nature or were “captivated by the beauty of nature” much more often than they had explicit feelings of “wondering awe” or were pausing “spellbound at the moment”; becoming “very quiet and devout” at certain places and thinking about all the things one is grateful about were experienced “in-between” (Table 2). A quite large fraction was seldom or never experiencing “feelings of wondering awe” (46%) or were pausing “spellbound in the moment” (40%).

**Table 2:** Expression of wondering awe indicators in the sample.

		Never (%)	Seldom (%)	Often (%)	Very often (%)	Mean score (0–3)
ED1	I have a feeling of great gratitude.	3.3	19.5	48.7	28.5	2.02±0.78
ED2	I have a feeling of wondering awe.	9.5	36.4	38.6	15.4	1.60±0.86
ED3	I still have learned to experience and value beauty.	1.1	8.7	51.9	38.3	2.27±0.66
ED4	I stop and am captivated by the beauty of nature.	1.8	14.7	44.8	38.7	2.20±0.75
ED5	I pause and stay spellbound at the moment.	4.2	35.6	41.5	18.8	1.75±0.80
ED6	In certain places I become very quiet and devout.	3.2	25.7	44.6	26.5	1.94±0.80
ED7	I stop and then think of so many things for which I am really grateful.	3.6	24.9	46.6	24.8	1.93±0.80

**Figure 1:**
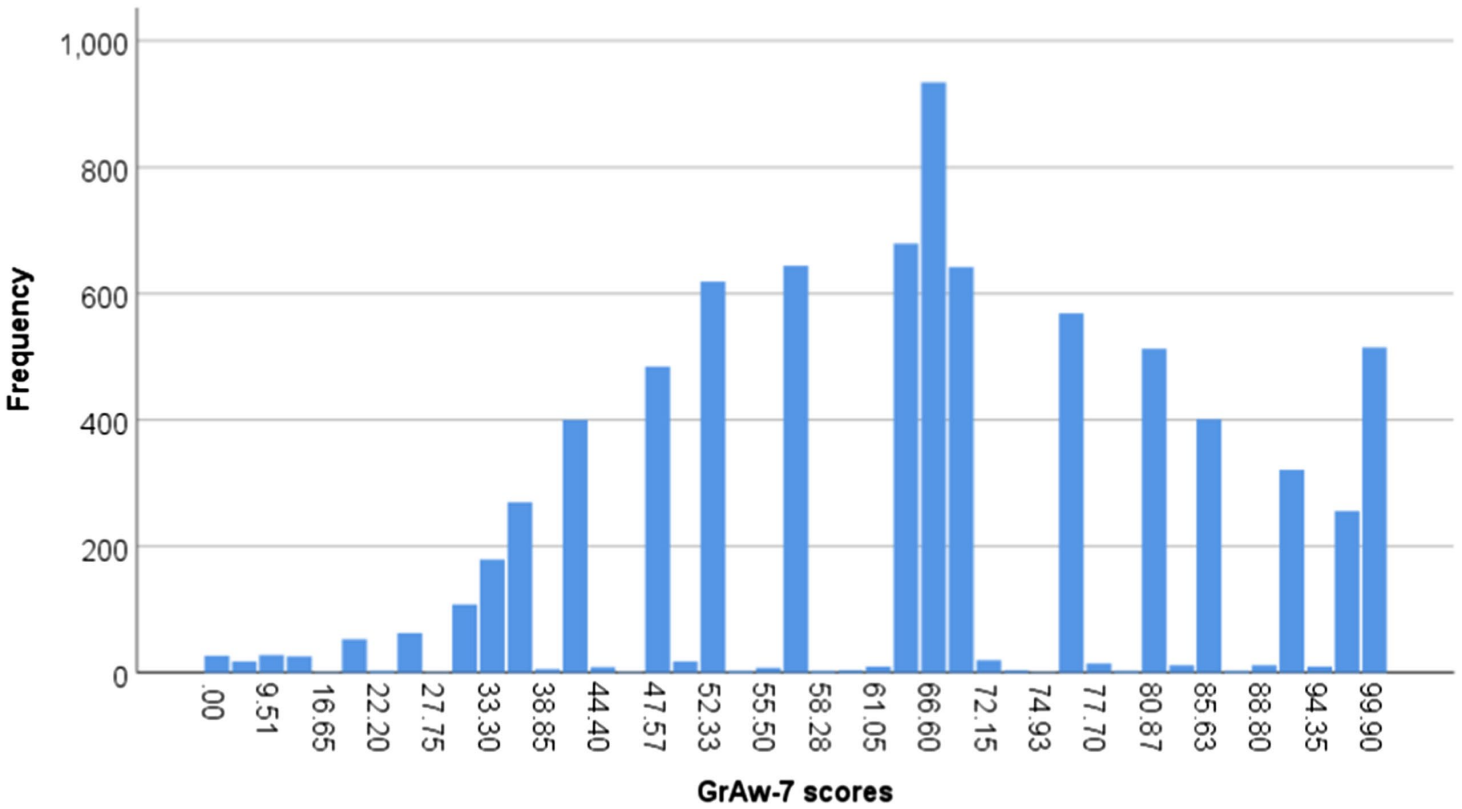
Range of GrAw-7 scores in the sample.

These seven items can be combined to one factor, the GrAw-7 scale, which has a good internal consistency also in this large sample (Cronbach’s alpha=0.88) and explains 58% of variance. The mean score of this factor is 65.3±19.7 (range 0–100; Figure 1). Here, 15% have scores<1 SD of the mean, indicating low GrAw-7 scores, and 19% >1 SD, indicating high GrAw-7 scores.

Women have significantly higher scores than men (67.6±19.3 vs. 61.3±19.9, *F*=184.0, *p*<0.0001; Cohen’s *d*=0.32). Also, the age cohorts differ significantly (*F*=101.4, *p*<0.0001), with the lowest GrAw-7 scores in the younger ones (<21years: 56.4±22.4) and the highest in the older ones (>70years: 72.7±17.2; Cohen’s *d*=0.80). Persons with a nominally Christian background have higher scores than those without (66.4±19.5 vs. 62.8±20.8), yet this difference is rather weak (Cohen’s *d* =0.18). However, persons with other religious affiliations had the highest GrAw-7 scores (70.6±18.3); compared to Christians the difference is weak, too (Cohen’s *d*=0.22). Thus, the religious orientation had a small but significant influence on the GrAw-7 scores (*F*=21.6, *p*<0.0001).

In a subsample of 5,370 persons, the GrAw-7 scores correlated moderately with participants’ wellbeing (WHO-5: r=0.36, *p*<0.0001) and in a subsample of 2,613 persons strongly with the perception of the Sacred in daily life (DSES-6: *r*=0.58, *p*<0.0001). Frequency of meditation and praying was assessed in 3,886 persons, and the GrAw-7 scores were moderately related with both spiritual practices (*r*=0.43 and 0.38, *p*<0.0001). As there is an obvious association between perception of the Sacred in life and frequency of spiritual practices, one may assume that particularly religious brothers and sisters (*n*=371) and yoga practitioners (*n*=833) with their specific lifestyles and spiritual practices will have higher GrAw-7 scores than the other persons. In fact, religious brothers and sisters, which are the oldest (mean age: 60.5±13.7years), had similarly high scores compared to yoga practitioners (72.5±14.8 vs. 74.5±16.5; Cohen’s *d*=0.13), which were much younger (mean age: 49.4±10.3years), while the scores of the other participants (mean age: 44.8±16.7) were significantly lower (63.8.6±19.9; *F*=141.3, *p*<0.0001).

Referring to the categorized frequency of Awe/Gratitude perceptions [low (<45.6), moderate (45.6–85.0), and high (>85.0)], those with low GrAw-7 scores had the lowest age, lowest wellbeing, and lowest spiritual practices, while those with high GrAw-7 scores were the oldest, had the highest wellbeing, and were more often meditating or praying (Table 3).

**Table 3:** GrAw-7 categories and relation to age, wellbeing, and spiritual practices.

		Age (years)	Wellbeing (WHO-5)	Frequency meditation	Frequency praying
Number		7,809	5,370	3,379	3,373
Awe/Gratitude perceptions					
Low (15%)	Mean	37.81	11.17	0.34	0.56
	SD	15.90	6.20	0.75	0.99
Moderate (66%)	Mean	46.36	14.49	1.01	1.39
	SD	16.06	5.13	1.15	1.30
High (19%)	Mean	51.20	17.45	1.85	2.01
	SD	15.36	4.69	1.19	1.26
All persons (100%)	Mean	45.99	14.46	0.99	1.30
	SD	16.38	5.55	1.17	1.31
*F* value		238.2	323.3	264.6	202.5
value of *p*		<0.0001	<0.0001	<0.0001	<0.0001

### Qualitative Approach

As shown in the empirical data, the frequency of the different indicators of perceived awe differs, with more frequent perceptions of beauty in nature in general than staying “spellbound” at the moment. This would mean that moments of fascination are experienced more often than the more specific perceptions of wondering awe. To analyze the different causes and triggers of a wide range of awe perceptions, a heterogeneous group of persons was invited to describe what caused them to perceive moments of wondering awe. Here, the statement of 82 responding persons was analyzed with qualitative content analysis techniques.

#### Triggers of Moments of Wondering Awe

**Figure 2:**
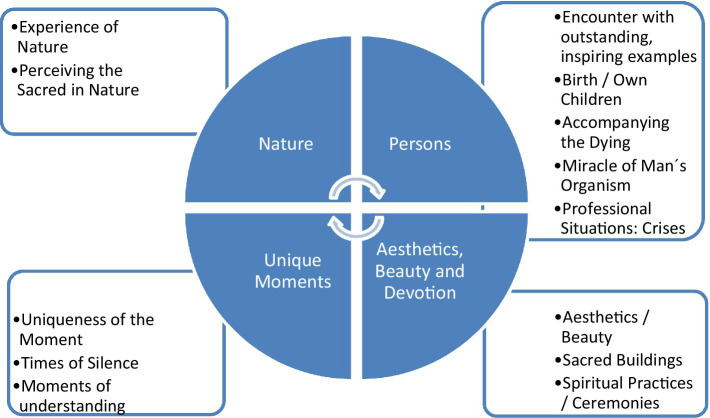
Main and subcategories of Awe triggers.

Identified triggers of awe can be related to four main categories, *Nature*, *Persons*, *Unique Moments*, and *Aesthetics*, *Beauty*, and *Devotion* (Figure 2). In the following, the main categories and their subcategories will be described and representative text passages that served as anchor quotation were added [with anonymized IDs of the person, gender (f for female and m for male), and age].

### Nature-Related Triggers

#### Experience of Nature

This topic has the most entries. Characteristic statements are as follows:

*“These moments usually happen when I am alone in nature which can be a small garden, a beach, watching a flower, or a landscape of unspoiled nature” (ID41, m, 56).*

More specific situations and observations related to nature were described, too:

*“Most of the time I feel that in or through nature. When I come to the pond one morning and see the empty hulls of the hatched royal dragonflies and then they buzz over the surface of the water. When I see the little frogs watering the flowers. To watch the young tits leave the box and flutter out into the world. The colors of a sunrise that are almost too beautiful to be true. The sea” (ID80, f, 50+).*

*“As a rule, these impressions are associated with nature in the broadest sense. It can be a beautiful landscape, or the observation of wild animals. I am an enthusiastic rider and when these impressions are still connected to my horse, i.e. when riding, then I often find the feeling overwhelming!” (ID19, f, 56).*

#### Perceiving the Sacred in Nature

Related to the aforementioned general topic, these perceptions were attribution by several persons to the Sacred in the whole creation, either stated directly or indirectly:

*“When I see the divine shimmering out of people/animals /plants/beings or the divine in their actions” (ID22, f, 29).*

*“When I take the ship to a German North Sea island in autumn and the weather is rough and stormy, I am amazed because of the greatness of God” (ID78, f, 73).*

*“I am amazed again and again by the creative work of nature and that of man. When I see how things have developed which are unbelievably creative, it often touches me deeply (ID21, m, 55).*

One woman interpreted her nature-related perceptions as a hint of God’s presence, particularly in depressive states.

*“In deep personal despair and fear, in the feeling of hopelessness, the perception of a robin, a sunrise, sun-drenched leaves of trees - small light events that have always filled me with astonished awe, because for me they are powerful signs of the no-ifs-and-buts validity of Jawhe’s almost incomprehensible promise [to be there for me]” (ID43, f, 65).*

One other woman stated that unique perceptions after her work with others reminded her that she is part of “something bigger,” without clearly referring it to something transcendent:

*“After intensive work - with others, moment of rest, ex. food, and the idea that I am embedded in something bigger*” *(ID119, m, 56).*

### Person-Related Triggers

#### Encounter With Outstanding, Inspiring Examples

Outstanding, inspiring people may also trigger perceptions of admiration or even awe. There are several statements that refer to this category. These persons can be impressive because of attitudes, behaviors, and actions, because of their life experiences, or because of outstanding skills:

*“I am full of amazement when a person acts ‘superhumanly’, such as when a Palestinian father releases an organ of his brain-dead son to the son of an Israeli father. I am in awe when people forgive circumstances (such as the murder of a family member) without a trace of retaliation. I am awesome when people behave altruistically by putting themselves in danger and saving people and animals. I am in awe of the wisdom of old people” (ID15, f, 44).*

Older persons and persons in difficult situations can cause moments of respect, admiration, and awe:

*“Also with older people who transmit wisdom or younger people in conversations” (ID41, m, 56).*

*“At patient encounters, when amazing human encounters take place, e.g. how someone deals with an extremely difficult illness: how someone thinks about the world and life and death” (ID163, f, 64).*

Important persons can also be members of the own family that helped to care for the children despite own burden:

*“Today, on my father’s birthday, I think of him with awe and gratitude. In the time of his loneliness as a (widower) he respected us, his growing daughters and helped to understand the maturing life” (ID82, f, 82).*

Also admiration of the unique skills of specific persons, either artists, musicians, or poets were categorized here:

*“With a 25-year-old pianist in the large concert hall, who played the hardest pieces by heart for* 2*h. The musician was still so young and played so confidently and at the same time so humbly, devoted, selflessly*… *out of the silence - no one who was listening interfered with coughing, etc.*”*(ID 101, f, 59).*

*“Perhaps most of all with music, for example when I see a musician I admire ‘live’ and feel that what he/she does is really great” (ID67; m, 69).*

*“It is also triggered when I hear beautiful music or singing. A great pleasure and astonished pause even with beautiful texts, especially poems, e.g.*, *Rilke” (ID115, f, 70).*

#### Birth/Own Children

The birth of a child was stated as a trigger of awe by some persons, not because of specific abilities or behaviors, but because of their simple presence and unique “wonderful” part of their life:

*“I was able to attend a birth. Amazed awe to hold a little new living being ‘human’ in your hands! You cannot do that, it’s a gift” (ID9, female, 71).*

*“Immediately after the birth of our first child, there was such an unexpected moment of ‘wondering awe’. This lasted extremely for a few minutes and then more and more weakly for several days. This feeling was also present at the birth of the second child, but weakened more quickly” (ID150, m, 54).*

Also living with the own children and attending their development causes moments of wondering awe:

*“Such moments are also familiar to me in everyday life with my child. Reaching for a toy for the first time, discovering your own toes and fingers, but also now (he’s now 8) watching my child, hearing how much wisdom, sincerity, clarity and compassion there is in this little person” (ID145, f, 37).*

#### Accompanying the Dying

Not only birth can be a trigger of awe, but also being with dying persons. These encounters were touching because the dying persons shared their experiences, and respondents were able to assist them:

*“In the face of dying/death - when a whole life is decided. Death as something great” (ID 99, m, 55).*

*“In the great moments of meeting patients, when they have invited me into their life, which was often enough determined by suffering, misery, despair, fear of death, and succeeded in helping them to give them a little bit of hope, discovered a little joy, a little gratitude in looking back at their life. This given premonition of being loved nonetheless has changed these people” (ID43, f, 65).*

Nevertheless, these moments are also a time of self-reflection, and one person was appreciating the confidence of specific persons and their acceptance of being dying:

*“Whenever I feel that death is in wait in a room, I am amazed at the YES of the person concerned. Then I ask myself: - Are you capable of this?” (ID82, f, 82).*

#### Miracle of Man’s Organism

This subcategory holds statements “from a distance” about man as a miracle in general. It is a matter of rather cognitive “wonder” how complex the creation and man specifically is:

*“The human organism - with all the intermeshing of the most varied of processes - to be aware of this causes me to be amazed again and again. Or the development of a little human being - getting up at some point, the emerging speaking and understanding of contexts” (ID130, f, 40).*

*“As a doctor, when I visualize what always happens so automatically and yet finely tuned unconsciously through our body, cells divide, food is digested” (ID132, f, 35).*

#### Professional Situations: Crises

Times of crisis triggered moments of awe in some respondents. These can be, for example, a matter of wondering surprise against own professional expectations in terms of healing processes:

*“If a child is born in an emergency, it has to be reanimated and all parameters are more pessimistic. And then, after a few days and repeatedly for weeks and months, the news comes that the child is healthy and well” (ID109, f, 57).*

One medical doctor described an own moment of crisis when she perceived simple moments of admiration as a hint that God is still at her site, even in times of inner darkness:

*“The astonished reverence was for this inviolability of the promise and the inventiveness of God founded in love, and that they [she refers to unexpected observations in nature] were also for me at the bottom of life. The question - why did you leave me - but it also implies knowing about you. And that was enough” (ID43, f. 65).*

### Unique Moments Related Triggers

#### Uniqueness of the Moment

Specific situations can trigger such feelings as described by some participants. Several triggers of the aforementioned category, particularly “Birth of the own child” or “Accompanying the dying” or “Crises,” could be attributed also to this category. A general statement addresses *“Moments in everyday life, looks, words” (ID162, f, 51)*, while there are also more specific situations. One statement indicates some kind of resonance between therapist and patient:

*“During rhythmic embrocations on patients when something like non-verbal communication is taking place. There is silence and calm in the room, and something begins to oscillate between patient and practitioner” (ID128, f, 53).*

#### Times of Silence

Specific times of silence at certain places and also during daily life activities were mentioned, either related to God or not specifically addressed:

*“Monastery - meeting God in silence” (ID28, f, 56).*

*“It can also happen spontaneously in everyday life, especially in times of silence or meditation” (ID32, m, 55).*

#### Moments of Understanding

This category refers to situations and moments which raised specific “unexpected” understanding or “insights” (i.e., of being “part of the creation,” gifts are not deserved but nevertheless given, “larger connections”) that thereby triggered moments of wondering awe. These “*insights*” (ID32, m, 55) refer to a perception of interconnectedness which may link this topic to the subcategory “Perceiving the Sacred in Creation,” too.

*“In the holm oak forest of a hermitage: silence, old trees, fog, just being in there as a person, and feeling, I am part of creation” (ID9, f, 71).*

*“The main reasons were the awareness of undeserved gifts. But also the awareness of being carried and chosen by God” (ID20, m, 47).*

*“Amazed awe occurs when I see larger connections in the world or in my life. Sometimes it happens as a spontaneous insight. Mostly when I read texts or take part in philosophical seminars” (ID32, m, 55).*

*“There is always amazement and thanks when I look at my life and so many unbelievable connections that emerged in it” (ID115, f, 70).*

### Aesthetics, Beauty, and Devotion

Some participants reported moments of admiration and even awe when they perceive music, poetry, ballet, or sacred buildings which have impressed or inspired them, or during specific ritual and ceremonies that have touched them. The sacred buildings are both, a place of admiration and veneration and a place to practice specific rituals.

#### Aesthetics and Beauty

Here, examples are categorized that go beyond simple admiration of artists as concrete persons, but refer to music or poetry itself which is touching:

*“When I walked into the Museum of Modern Art in New York and there was a real Matisse hanging in front of my eyes in real size and colors. To really see this in REAL was overwhelming” (ID23, m, 69).*

*“The incredible beauty of a Bach oratorio. The deep touch that I feel when I hear the Brahms requiem and even more when I sing it. Unexpected changes in harmony that completely surprise me and open completely new doors to the piece [of music] and maybe also to myself. A poem that uses language in such a way that the old words suddenly create something completely new. When language can surprise me, leave the usual path and create something new with inner contexts or its sound and rhythm that is unexpectedly beautiful. Dance. Mainly ballet or artistry” (ID80, w, 55+).*

#### Sacred Buildings

Specific sacred buildings were mentioned either in terms of the beauty of the building itself or its effects on the person. These can be related to perceptions of connectedness, too. However, it can be also an aesthetical experience of inspiring peace, calm and beauty.

*“When I came to the Sagrada Familia in Barcelona and was amazed at the format, the colors and the naturalness of the shapes” (ID23, m, 69).*

*“Large church buildings (feeling of “holiness,” respect for centuries-old tradition, feeling of belonging to a large community [of Christians])” (ID18, f, 38).*

*“Cathedrals especially when they are embedded in the quiet of a landscape. And the sacred can be felt far beyond the narrow confessional references” (ID157, f, 60).*

#### Spiritual Practices/Ceremonies

Depending on the spiritual background, specific spiritual/religious rituals were mentioned that have triggered feelings of awe by three persons. In case of a yoga practitioner, this feeling was also related to remembering a unique teacher of his tradition, in terms of devotion. However, in that respective situation, it was the ritual which triggers the devotion of the school’s guru (his picture is placed at the altar).

*“Every time in Eucharistic adoration. When receiving the sacraments” (ID20, m, 47).*

*“In front of an altar during a satsang [spiritual ceremony with chantings] while looking at a picture of Swami Sivananda [the deceased guru of this yoga school] during a puja [ritual of veneration]; in deep meditation” (ID16, m, 56).*

## Discussion

Findings of the empirical part of this study underline that the frequency of Awe/Gratitude perceptions is varying strongly. Persons with low Awe/Gratitude perceptions were the youngest and had the lowest wellbeing in the sample, while those with high Awe/Gratitude perceptions were the oldest and had the highest wellbeing. Those who were experiencing awe only rarely (low scores) have wellbeing scores that would indicate depressive states (WHO-5 scores<13). The causal pathways of this association are unclear. Both concurrent pathways might be true: Persons with low wellbeing (or depressive states) experience awe less intensive, while persons who are unable to stop for moments of wondering awe (because they are too busy) might be “happy” but not satisfied with their life. Such associations were already described (Büssing et al., 2020a, 2021e), but not their causality. Although women scored significantly higher than men, gender had only a weak influence on awe perceptions, while the negative age-related effect could be attributed either to different life experiences in older persons and thus higher sensitivity for “important moments” in life or to another focus in the life of younger people, for whom other things in life are more important than for the elderly. As shown in previous studies, too (Büssing et al., 2020a, 2021a), GrAw-7 scores were moderately related to participants’ frequency of meditation and/praying, indicating that these spiritual practices may sensitize the awareness for moments of wondering awe. In fact, religious brothers and sisters and also yoga practitioners with their specific lifestyles and their regular spiritual practices had the highest GrAw-7 scores. This would indicate that the perception of awe could be either trained by spiritual practices or is part of this spiritual development process during the practice. This could be seen as a matter of embodied mindfulness resulting in feelings of gratitude, reconnection with own values and spiritual beliefs, too (Poletti et al., 2019). Therefore, these practices could be a relevant resource to perceive the world around and the given situations more mindfully aware.

During the COVID-19 pandemic, the perception of Awe/Gratitude was related best with positively perceived changes in terms of Nature/Silence/Contemplation and Relationships, while it was only marginally associated with participants’ perceived burden – and is thus not a buffer against burdening situations (Büssing et al., 2021a). Interestingly, Awe/Gratitude mediated the effect of Nature/Silence/Contemplation as a predictor on participants’ wellbeing as an outcome (Büssing et al., 2021a). Thus, the ability to remain in contemplative quietness and silence and to mindfully perceive nature contributes to a person’s wellbeing, which is mediated by the ability to experience Awe/Gratitude. During the COVID-19 pandemic, it was observed that the positively perceived changes in terms of Nature/Silence/Contemplation started to slowly decrease along with Awe/Gratitude after the first lockdown and the summer period and strongly declined during the second lockdown with its burdening restrictions, while wellbeing and stressors remained stable in the first phase and changed during the second lockdown. This would indicate that the decline of Awe/Gratitude preceded the decline of wellbeing; yet, Awe/Gratitude predicted only 9% of participants’ wellbeing variance (Büssing et al., 2021a) and is thus not the main relevant influence.

During the pandemic, GrAw-7 scores were much lower in tumor patients (mean score 57.4±20.2; Büssing et al., 2020a) compared to non-diseased persons (66.8±17.9; Büssing et al., 2020b). At that time, 35% of tumor patients (Büssing et al., 2020a) and 30% of non-diseased persons (Büssing et al., 2020b) had wellbeing scores that would indicate rather depressive states (WHO-5 scores<13). Both variables are connected, but the causal direction is unclear. In that sample of tumor patients, Awe/Gratitude was weakly related to the presence component of meaning in life directly after the first lockdown (MLQ) and moderately at the start of the second wave of the pandemic – and having meaning in life contributed to their wellbeing (Büssing et al., 2020a, 2021e). However, in tumor patients meaning in life predicted their wellbeing only to some extend (*R*^2^=0.12), but not Awe/Gratitude. It seems that the perception of Awe/Gratitude is a relevant dimension related to a person’s meaning in life and prosocial behavior, while it is not a direct aspect of their quality of life. It is rather an ability to nevertheless perceive the positive aspects in life – despite restrictions (in terms of the COVID-19 pandemic or illness).

As nature-related perceptions were the easiest approach to perceive moments of wondering awe, these are primarily addressed in the research instruments. However, there might be other triggers of awe that could be of relevance for specific persons and situation. Here, qualitative approaches are suited to address these further triggers and causes and also individual interpretations. The perception of beauty in nature was stated by most participants in the qualitative approach, and it was the most frequent theme in the study of Yaden et al. (2019), too. Yet, the qualitative analyzes revealed a wider range of awe triggers, encompassing, for example, Experience of nature, Perceiving the sacred in nature, Encounter with outstanding, inspiring people, Birth/own children, Accompanying the dying, Professional situations/Crises, Times of silence, Art, Music, and Poetry, Sacred buildings, and Spiritual practices/ceremonies. These can be assigned to four main categories, (1) Nature, (2) Persons, (3) Unique Moments, and (4) Aesthetics, Beauty, and Devotion, with their respective subcategories. However, some of the perceptions related to skilled persons may be more a matter of admiration than wondering awe, while in the case of the yoga practitioner, it seems to be a matter of devotion toward the yoga lineage’s guru during a spiritual ceremony. In the empirical study of Yaden et al. (2019), we find some shared themes, that is, persons with “Great skills,” “Great virtue,” or “Powerful leaders” and also “Music,” “Art,” and “Buildings or Monument” (Yaden et al., 2019); their theme “Grand theory or Idea” may find some similarities in our subcategory “Moments of understanding”; their theme “Encounter with God” cannot easily be related to one of our subcategories. They further stated that some persons mentioned “childbirth as a trigger for intense awe experiences,” and this topic was found in our study, too. However, in our qualitative approach, several others topics appeared which are not found in the categories mentioned by Yaden et al. (2019), that is, “Accompanying the Dying” or “Crises” in life, the experience of a moment’s “Uniqueness” and also “Times of Silence,” further “Perceiving the Sacred in Nature,” and additionally “Spiritual practices/ceremonies.” It is thus obvious that the range of awe triggers is wide and that there are different chances to be “touched” and moved by these encounters, also during times of crisis. This aspect is of relevance as this would argue against the suggestion that wellbeing is the necessary prerequisite of the ability to perceive moments of wonder and awe. Indeed, in tumor patients, depressive symptoms were negatively related to their trait mindfulness, and this link was mediated by their perceptions of meaning in life (Hsieh et al., 2021). Although one may disagree, in terms of art *per se* and performing artists, it was suggested that these are appreciated or admired because of their skills and that art is a starting point to “wonder,” but it is not necessarily a matter of awe (Fingerhut and Prinz, 2018). Some of the mentioned perceptions may have no relevant effects on the life of the respective persons (the small moments of appreciation or fascination), while other perceptions may be more profound and will result in changes of a person’s attitudes and behaviors. Gallagher et al. (2015) described that astronauts “have reported experiences that are deeply aesthetic, spiritual, or sometimes religious.” These perceptions are of course extraordinary and thus go beyond everyday experiences. In consequence, rather outstanding experiences and perceptions than small moments of fascination may trigger processes of change in terms of a person’s values, attitudes, and behaviors and can also result in processes of “spiritual transformation” (Keltner and Haidt, 2003; Ironson and Kremer, 2009; Kremer and Ironson, 2009; Cohen et al., 2010; Büssing et al., 2018b; Penman, 2021). Gallagher et al. (2015) stated that the above-described astronauts’ experiences resulted in higher spiritual sensitivity, that they were “more attuned ecologically or ethically after their return to Earth,” and further that “these experiences have been life transforming” for some of them. In this context, the “spiritual” is not meant as something beyond our sensory experiences (transcendent), but as something immanent. Nevertheless, some persons may interpret these experiences in the context of their worldview and religious faith, as it was reported particularly by religious persons in in the qualitative analyses.

Further outcomes of such processes of change can be lower stress perception, mental stability, better coping strategies, finding new meaning in life, optimism, substance-abuse recovery, and increased spirituality (Ironson and Kremer, 2009; Kremer and Ironson, 2009). As the perception of Awe/Gratitude is also related to prosocial behaviors (Piff et al., 2015) and mindful encounter and care for the environment and persons in need (Büssing et al., 2009, 2013, 2014), similarly to the findings of Gallagher et al. (2015), this would be a further argument to foster this resource, as it contributes to a person’s meaning in life and wellbeing (Rudd et al., 2012; Krause and Hayward, 2015; Zhao et al., 2019; Aschoff, 2021; Büssing et al., 2021a).

### Limitations

With respect to the empirical approach, different data sets using the GrAw-7 scale were combined, while, however, not all utilize the same additional instruments (WHO-5, DSES-6 or frequency of spiritual practices). Therefore, the number of participants is not the same for the respective correlation analyses. As responding to the DSES-6 requires a theistic religious belief, the scale was used only to underline convergent validity of the GrAw-7 scale (which does not require belief in God) in a subsamples of participants. Further, the data are cross-sectional, and thus, the causality of interactions remains unclear. Despite the relatively large sample size, we cannot guarantee that the data are representative for the general German population. Particularly, the two specific subsamples of religious brothers and sisters and of yoga practitioners were exclusively recruited as “positive perception” samples which are not representative for the general population.

With respect to the qualitative approach, the participants were invited to respond by email. Although it was assured that the responding emails were directly deleted and no identifying data were recorded, some may have been reluctant to send their responses back. One may further assume that particularly persons have responded who have an interest to state their usually positive experiences, while those who may assume that their experiences are either “too small” to report, not important enough or even negative might not have responded. Therefore, the number of participants was raised in order to achieve a larger set of different awe perceptions. Further, it cannot be excluded that persons from other cultural or religious societies may perceive moments of wondering awe differently than the German sample and that they may state other triggers, too.

## Conclusion

What is the “spiritual” aspect of perceiving moments of wondering awe? First, touching experiences that transcend the conventional experiences can induce processes of inner change (Cohen et al., 2010). These transformation processes can be small or more profound; the small ones could be seen in terms of mindful awareness without any relevant consequences, while the more deep impact perceptions may trigger new insights, attitudes, and behaviors. For both perceptions, causes or triggers are required, and these might be very heterogeneous and differentially perceived by different persons. As shown in this analysis, these triggers may be related to *Nature*, *Persons*, *Unique Moments*, or *Aesthetics*, *Beauty*, and *Devotion*. Some of them have a transcendent (non-materialistic) connotation, and others are concrete. The experiential and perceptive aspect might be crucial instead of adopting inherited experiences of others or adopting religious concepts for which no personal experience is available (belief systems). Here, the concept of transcendentality could enhance the view (Khachouf et al., 2013), as mindful states are first a matter of awareness that does not require external stimuli or a “perceptor,” but are nevertheless perceived on the other hand. Regular contemplative practices, which facilitate awe perceptions, may change “the provisional quality of our conceptual structure,” as suggested by Khachouf et al. (2013) and may thus change the behaviors and creativity. Second, these perceptions of wondering awe imply an openness to new experiences that may “emotionally touch” a person. Whether this experience is interpreted in the light of specific religious or non-religious frameworks and individual mindsets indicates whether it is seen as “trans-personal” or not. More relevant is the consequence of being “touched” in this way and by which trigger and how it is contextualized, as these factors influence putative spiritual transformation processes resulting in more conscious interactions with others and the world around, more caring and compassionate behaviors, new meaning in life, and a deepening of specific practices or rituals (i.e., mediation, praying) that are considered to connect to transcendent or immanent resources of hope (Büssing et al., 2018b; Büssing, 2019). With respect to these moments of wondering awe and subsequent feelings of gratitude, it is important to underline that this experiential aspect of spirituality is perceived by both, religious and non-religious persons – although stronger by persons with a dedicated religious or spiritual lifestyle and related practices. Nevertheless, also non-religious persons are “touched” by the beauty of nature and may perceive the “Sacred” in specific situations and moments, and also, these persons may change in terms of transformation processes. Yet, this does not exclude the possibility that religious persons interpret the same perceptions of wondering awe in terms of their belief system (i.e., that God is present in all things and can thus be experienced in nature, and also in other persons), and thus, the GrAw-7 scores are strongly related to the perception of the Sacred (God) in their life. Nevertheless, Gallagher et al. (2015) underlined that “awe and wonder are experiences that transcend religion, culture, politics.” This requires openness and an ability to go “outside of ourselves to explain or understand our senses of awe and wonder” (Gallagher et al., 2015).

**Figure 3:**
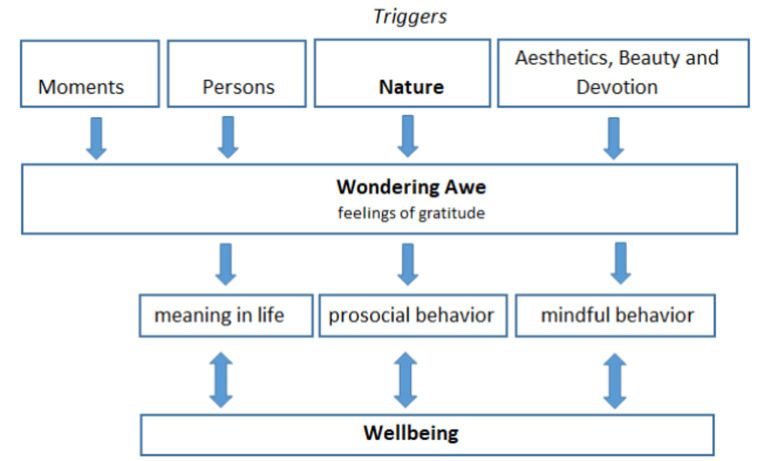
Putative pathways of awe triggers and related outcomes.

For healthcare professionals, this indicator of spiritual sensitivity could be an approach to implement the underlying ability to perceive the nevertheless beautiful and unique moments even in difficult life situations, both in their patients and in themselves, in terms of a coping process and to draw attention to what is still valuable and special in life. Mindful approaches, such as yoga and meditation, but also autobiographical narratives, art, and writing, could be useful. Perceiving moments of wondering awe will not directly influence a person’s wellbeing, but probably in terms of interaction processes that enroll different interacting variables (Figure 3).

## Data Availability Statement

According to the data protection regulations, the dataset cannot be made publicly available. Data are however available upon reasonable request.

## Ethics Statement

Ethical review and approval was not required for the study on healthy human participants in accordance with the local legislation and institutional requirements. Participants were informed about the study purposes, guaranteed confidentially, and they consented to participate by filling the anonymous questionnaires, which were applied in most cases online. Neither identifying personal details nor IP addresses were recorded to guarantee anonymity.

## Author Contributions

AB designed the study, set up the online survey, undertook all analyses, and wrote the manuscript.

## Conflict of Interest

The author declares that the research was conducted in the absence of any commercial or financial relationships that could be seen as a potential conflict of interest.

## Publisher’s Note

All claims expressed in this article are solely those of the authors and do not necessarily represent those of their affiliated organizations, or those of the publisher, the editors and the reviewers. Any product that may be evaluated in this article, or claim that may be made by its manufacturer, is not guaranteed or endorsed by the publisher.
